# Supplementary figures and images for: Two strains of Toscana virus show different virulence and replication capacity in mice and cell culture models
Source: Virulence. 2025 Jul 22;16(1):2535470. doi: 10.1080/21505594.2025.2535470 (PMC12296115; doi:10.1080/21505594.2025.2535470)

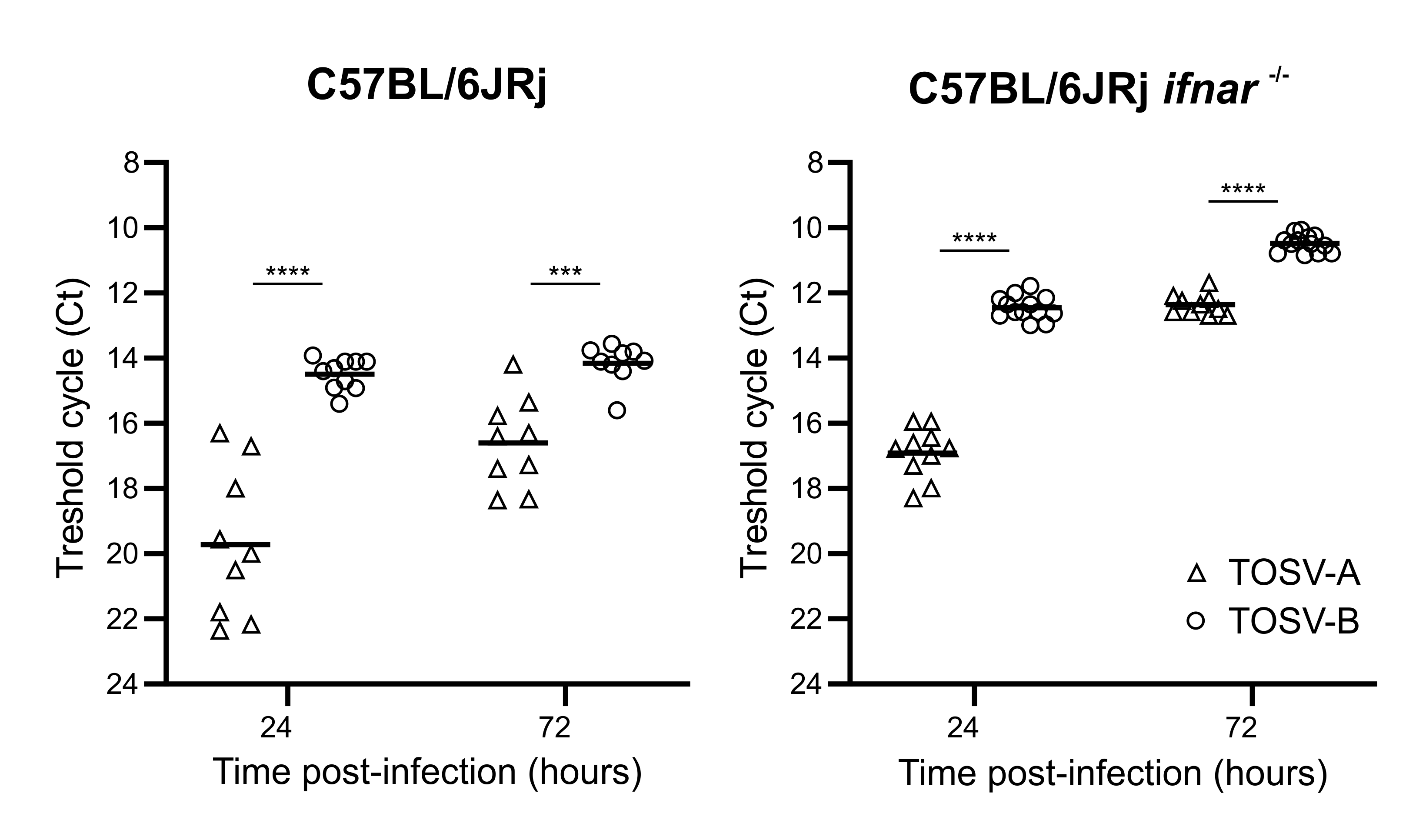

Supplement: QVIR-2024-0821.R1 - Figure S2.tif [file KVIR_A_2535470_SM6306.tif]

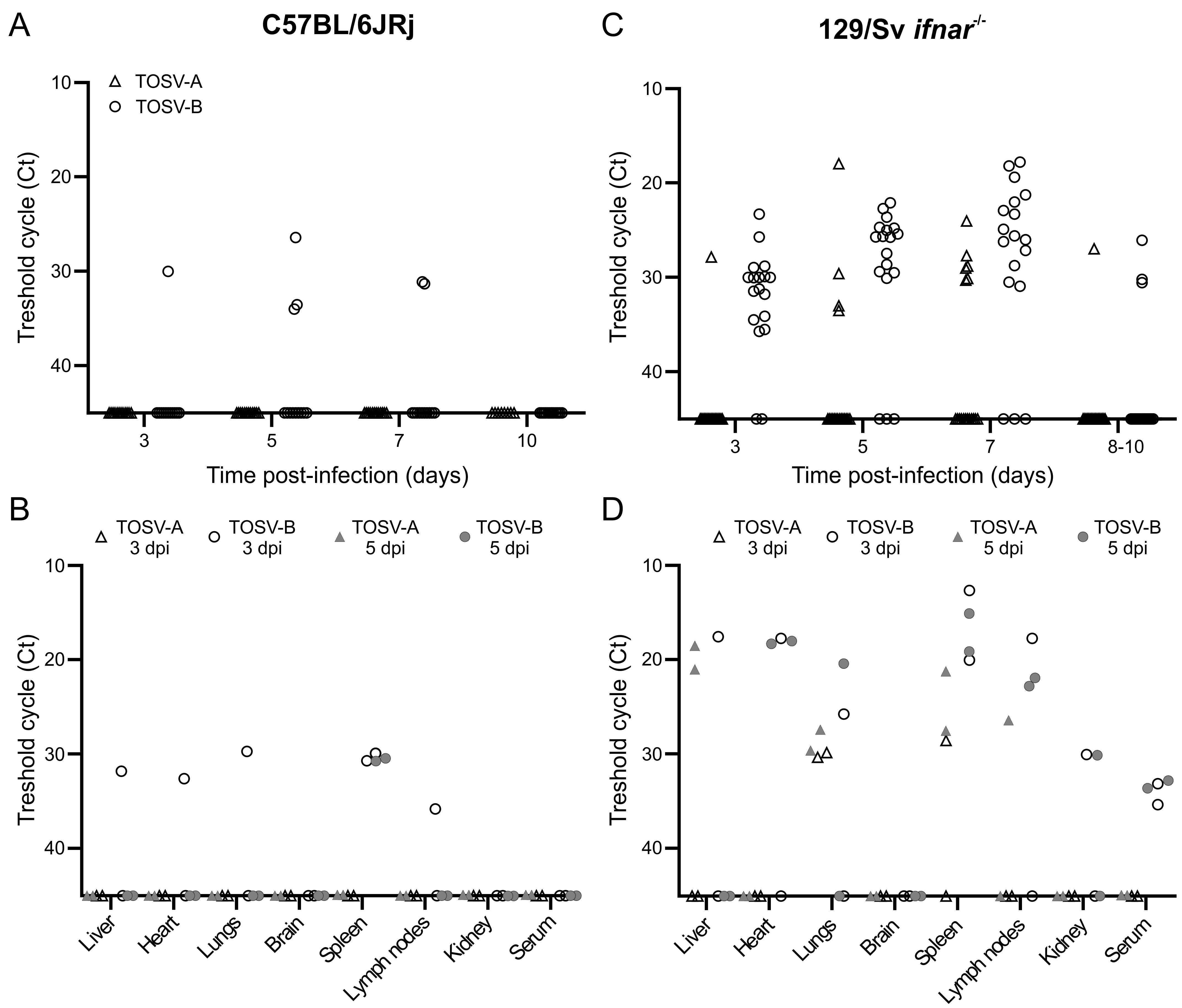

Supplement: QVIR-2024-0821.R1 - Figure S1.tif [file KVIR_A_2535470_SM6304.tif]
