# Supplementary material for: Two strains of Toscana virus show different virulence and replication capacity in mice and cell culture models
Source: Virulence. 2025 Jul 22;16(1):2535470. doi: 10.1080/21505594.2025.2535470 (PMC12296115; doi:10.1080/21505594.2025.2535470)
Supplement: Roy_et_al_Virulence_Supplemental_Files_R1.docx [file KVIR_A_2535470_SM6305.docx]

Two strains of Toscana virus show different virulence and replication capacity in mice and cell culture models.

Marlène Roy^1¶^, Sandra Lacôte^2¶^, Sophie Desloire^1^, Adrien Thiesson^1^, Coralie Pulido^3^, Noémie Aurine^4^, Cyrille Mathieu^5^, Bertrand Pain^4^, Philippe Marianneau^2^, Frédérick Arnaud^1*^, Maxime Ratinier^1^*

^1^ IVPC UMR754, EPHE, Université PSL, INRAE, Universite Claude Bernard Lyon 1, F-69007 Lyon, France

^2^ ANSES, Virology Unit, F-69007 Lyon, France

^3^ ANSES, Laboratoire de Lyon, Plateforme d'Expérimentation Animale, 69007 Lyon, France

^4^ Université Lyon 1, INSERM, INRAE, Stem Cell and Brain Research Institute, U1208, USC1361, F-69675 Bron, France

^5^ CIRI, Centre International de Recherche en Infectiologie, Team Neuro-Invasion, TROpism and VIRal Encephalitis, INSERM, U1111, CNRS, UMR5308, Universite Claude Bernard Lyon 1, Ecole Normale Supérieure de Lyon, 69007 Lyon, France

^¶^ These authors contributed equally to this work

* Corresponding authors

E-mail : [maxime.ratinier@univ-lyon1.fr](mailto:maxime.ratinier@univ-lyon1.fr); [frederick.arnaud@univ-lyon1.fr](mailto:frederick.arnaud@univ-lyon1.fr)

**Key words**: Toscana virus, TOSV, mouse model, pathogenesis, genetic lineage

**Supporting information**


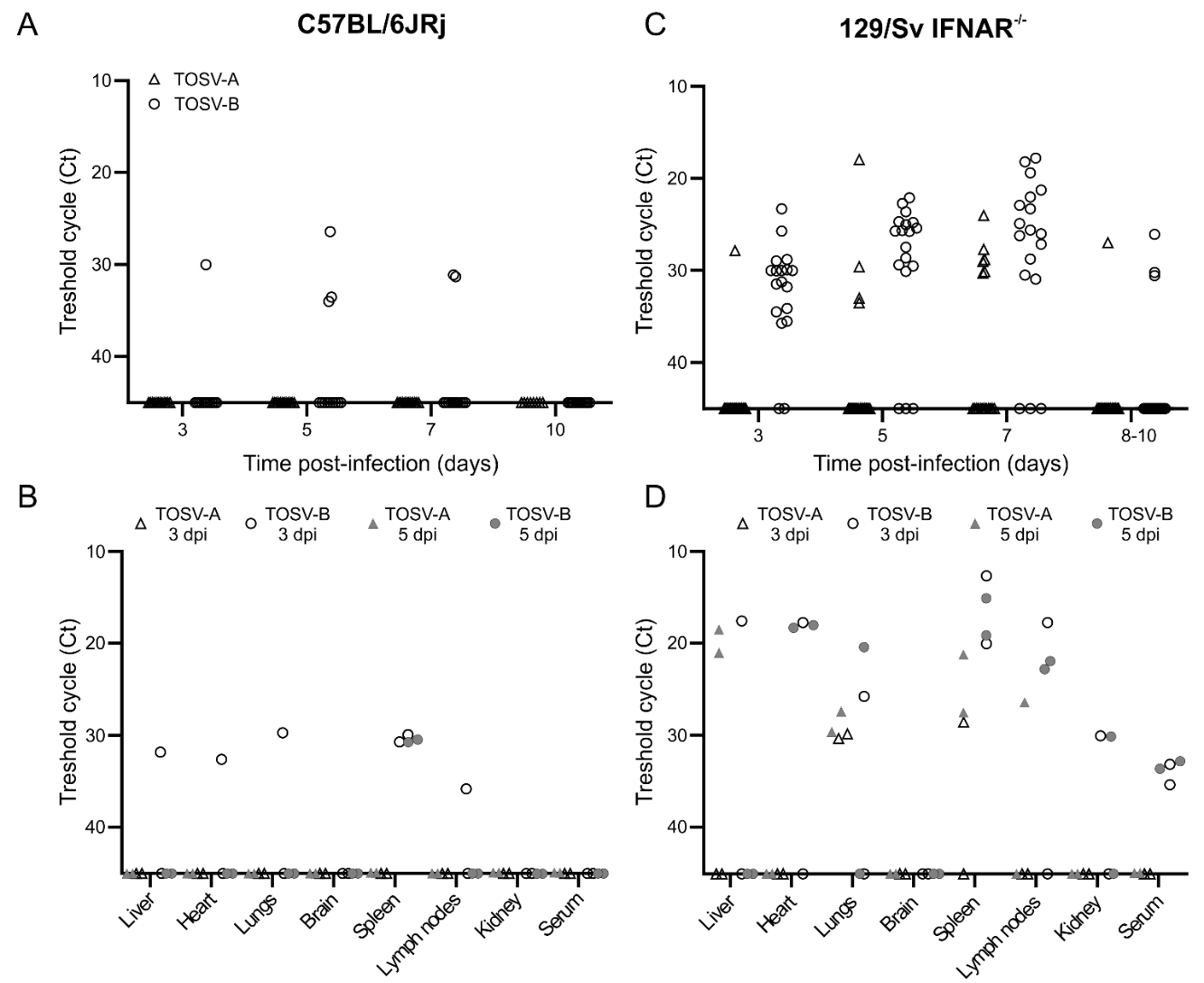


**Figure S1: Levels of TOSV RNA in sera and organs of C57BL/6JRj and 129/Sv *ifnar*^-/-^ mice.** Detection of TOSV RNA in the sera (RNAemia) of C57BL/6JRj mice (A) and 129/Sv *ifnar* ^-/-^ mice (C). Sera were collected at 3, 5, 7, and 10 dpi. Levels of viral RNA in sera of infected mice were measured by RT-qPCR targeting segment S. Cycle threshold (Ct) values are represented. Levels of viral RNA in the organs and serum of C57BL/6JRj mice (B) and 129/Sv *ifnar* ^-/-^ mice (D) at 3 and 5 dpi. Mice, subcutaneously infected with 10^3^ PFU of either TOSV-A or TOSV-B, were euthanized at either 3 (white triangles and circles) or 5 dpi (grey triangles and circles). Organs and sera were collected and the presence of TOSV RNA was assessed using RT-qPCR targeting segment S. Ct values are represented. .


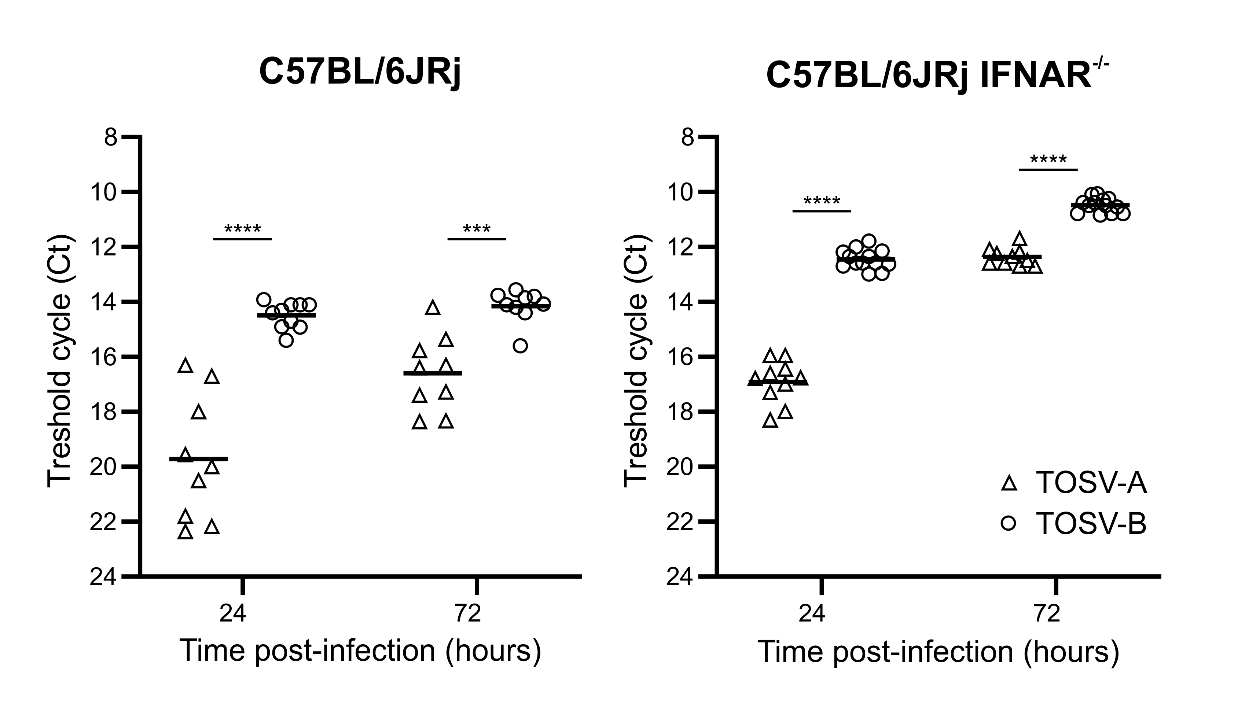


**Figure S2: Levels of TOSV RNA in OBC.** Each OBC, obtained from either C57BL/6JRj or C57BL/6JRj *ifnar* ^-/-^ mice, was infected with 10^3^ PFU of either TOSV-A or TOSV-B. Levels of viral RNA in each OBC were measured by RT-qPCR targeting segment S. Individual Ct and mean values are represented. Comparisons of Ct values at each time point between TOSV-A and TOSV-B were carried out using the Mann-Whitney test; P < 0.001 (***) and P < 0.0001 (****).
